# Detection of risk clusters for deaths due to tuberculosis specifically in areas of southern Brazil where the disease was supposedly a non-problem

**DOI:** 10.1186/s12879-019-4263-1

**Published:** 2019-07-17

**Authors:** Luana Seles Alves, Danielle Talita dos Santos, Marcos Augusto Moraes Arcoverde, Thais Zamboni Berra, Luiz Henrique Arroyo, Antônio Carlos Vieira Ramos, Ivaneliza Simionato de Assis, Ana Angélica Rêgo de Queiroz, Jonas Boldini Alonso, Josilene Dália Alves, Marcela Paschoal Popolin, Mellina Yamamura, Juliane de Almeida Crispim, Elma Mathias Dessunti, Pedro Fredemir Palha, Francisco Chiaraval-Neto, Carla Nunes, Ricardo Alexandre Arcêncio

**Affiliations:** 1 Nursing Graduate Program in Public Health Nursing, University of São Paulo at Ribeirão Preto Nursing College, 3900 Avenida dos Bandeirantes, São Paulo, Brazil; 2 Inter-institutions Doctoral Program in Nursing, University of São Paulo at Ribeirão Preto Nursing College, São Paulo, Brazil; 3 Public Health, Natal, Rio Grande do Norte Brazil; 4 University of São Paulo at Ribeirão Preto Nursing College, São Paulo, Brazil; 5 Department of Nursing, Fasipe School, Mato Grosso, Brazil; 6 Department of Nursing, Londrina State University, Londrina, Paraná Brazil; 7 Department of Epidemiology, School of Public Health, University of São Paulo, São Paulo, São Paulo Brazil; 8 National School of Public Health, Nova University of Lisbon, Lisboa, Portugal; 9 Maternal-Infant and Public Health Nursing Department, University of São Paulo at Ribeirão Preto College of Nursing, Av dos Bandeirantes 3900, Ribeirão Preto, São Paulo 14040-902 Brazil

**Keywords:** Tuberculosis, Death, Spatial analysis, Isotonic regression, Scan statistics, Cluster detection

## Abstract

**Background:**

Tuberculosis (TB) is the infectious disease that kills the most people worldwide. The use of geoepidemiological techniques to demonstrate the dynamics of the disease in vulnerable communities is essential for its control. Thus, this study aimed to identify risk clusters for TB deaths and their variation over time.

**Methods:**

This ecological study considered cases of TB deaths in residents of Londrina, Brazil between 2008 and 2015. We used standard, isotonic scan statistics for the detection of spatial risk clusters. The Poisson discrete model was adopted with the high and low rates option used for 10, 30 and 50% of the population at risk, with circular format windows and 999 replications considered the maximum cluster size. Getis-Ord Gi* (Gi*) statistics were used to diagnose hotspot areas for TB mortality. Kernel density was used to identify whether the clusters changed over time.

**Results:**

For the standard version, spatial risk clusters for 10, 30 and 50% of the exposed population were 4.9 (95% CI 2.6–9.4), 3.2 (95% CI: 2.1–5.7) and 3.2 (95% CI: 2.1–5.7), respectively. For the isotonic spatial statistics, the risk clusters for 10, 30 and 50% of the exposed population were 2.8 (95% CI: 1.5–5.1), 2.7 (95% CI: 1.6–4.4), 2.2 (95% CI: 1.4–3.9), respectively. All risk clusters were located in the eastern and northern regions of the municipality. Additionally, through Gi*, hotspot areas were identified in the eastern and western regions.

**Conclusions:**

There were important risk areas for tuberculosis mortality in the eastern and northern regions of the municipality. Risk clusters for tuberculosis deaths were observed in areas where TB mortality was supposedly a non-problem. The isotonic and Gi* statistics were more sensitive for the detection of clusters in areas with a low number of cases; however, their applicability in public health is still restricted.

## Background

Tuberculosis (TB) is an ancient disease that remains a serious public health problem worldwide, affecting 30 countries that account for 87% of TB cases. The disease is among the infectious disease that kills the most people worldwide, more than HIV and malaria [1]. The World Health Organization (WHO) launched the ‘End TB’ strategy in 2014, aiming to achieve the elimination of TB by 2050 (< 1 case per 100,000 people), and to also reduce TB mortality by 95% by 2035; both goals are a large challenge in developing countries, such as Brazil [2].

In 2017, Brazil had an incidence rate of 44 cases per 100,000 inhabitants and a mortality rate of 2.4 per 100,000 inhabitants, with a treatment success rate of 72% among the patients monitored in 2016; this result falls short of the WHO recommendations [1]. Recently, the ‘Brazil free of tuberculosis’ strategy was adopted, which aims to improve access to diagnosis and quality treatment in order to achieve the goals defined by the WHO [2]. The strategy is also based on three pillars: integrated and patient-centred prevention and care; bold policies and supportive systems; and the intensification of research and innovation [3].

Currently, government predictions outline that Brazil will achieve the goal of reducing mortality by 2035 at the national level [2]; however, it is unlikely that this will happen at the subnational levels, i.e. states or municipalities. This is due to Brazil being one of the largest countries in the Americas, occupying almost 20.8% of the hemisphere and 47.3% of South America, making it difficult to reduce the mortality rate homogeneously throughout all regions [2].

Studying the subunits of the country and thus an approximation of local health systems is relevant in order to highlight the trends and impact of tuberculosis mortality in these scenarios [4]. Mortality due to TB is defined as a socially determined event, marked mainly by social inequalities related to income, schooling, housing conditions, labour conditions, the weakness of health services (delay in diagnosis, poor diagnosis and treatment) and the absence of social politics and social protection. The risk of deaths is influenced by these factors, which range between areas in a same city, county or country [5, 6], therefore measuring the spatial risk to which given communities are exposed can contribute to the guidance of public policies and strategic actions [7].

This situation is not static. On the contrary, it is very dynamic and varies between the regions according to the availability of resources in terms of health, social investment, urbanisation, immigration, and modes of social organisation; therefore, the risk of disease and its impact (fatality) are influenced by these social dynamics [5]. The literature indicates that, in certain territories, deaths from TB are more prevalent in areas with social problems and a lack of assistance, however, there are few studies that show this, which is important for addressing the problem [6]. Therefore, measuring the spatial risk to which given communities are exposed can contribute to the guidance of public policies and the adoption of directed and focused health measures.

Tuberculosis is a low frequency event compared to diseases such as hypertension, diabetes and malaria, among others; it affects very specific and/or minor populations or groups. Therefore, because traditional techniques, such as scan statistics, require a greater number of event occurrences, their use may lead to erroneous inferences of the non-existence of the death risk of TB in a vulnerable community when it is in fact present and imminent.

The literature has a wide variety of definitions and methodological approaches for identifying clusters [8]; various methods are compared, contrasted and matched, aiming to overcome the issue of unknown and underdiagnosed clusters and to ensure the validity, sensitivity and accuracy of the studies [9].

Through a literature review of TB mortality risk clusters, only three articles were identified in Brazil [6, 8, 9], however all of these articles applied standard scan statistics. Isotonic spatial scan statistics and Getis-Ord Gi* statistics (Gi*) can be used complementarily to increase the chances of identifying a cluster, mainly in areas where the event is more rare. Although these statistics are interesting resources to identify spatial risk clusters in the case of rare phenomena, their application in the area of geoepidemiology is still very limited. Thus, this study aimed to identify risk clusters for TB deaths, specifically in areas where TB supposedly does not seem to be a problem.

## Methods

### Study design and setting

**Fig. 1:**
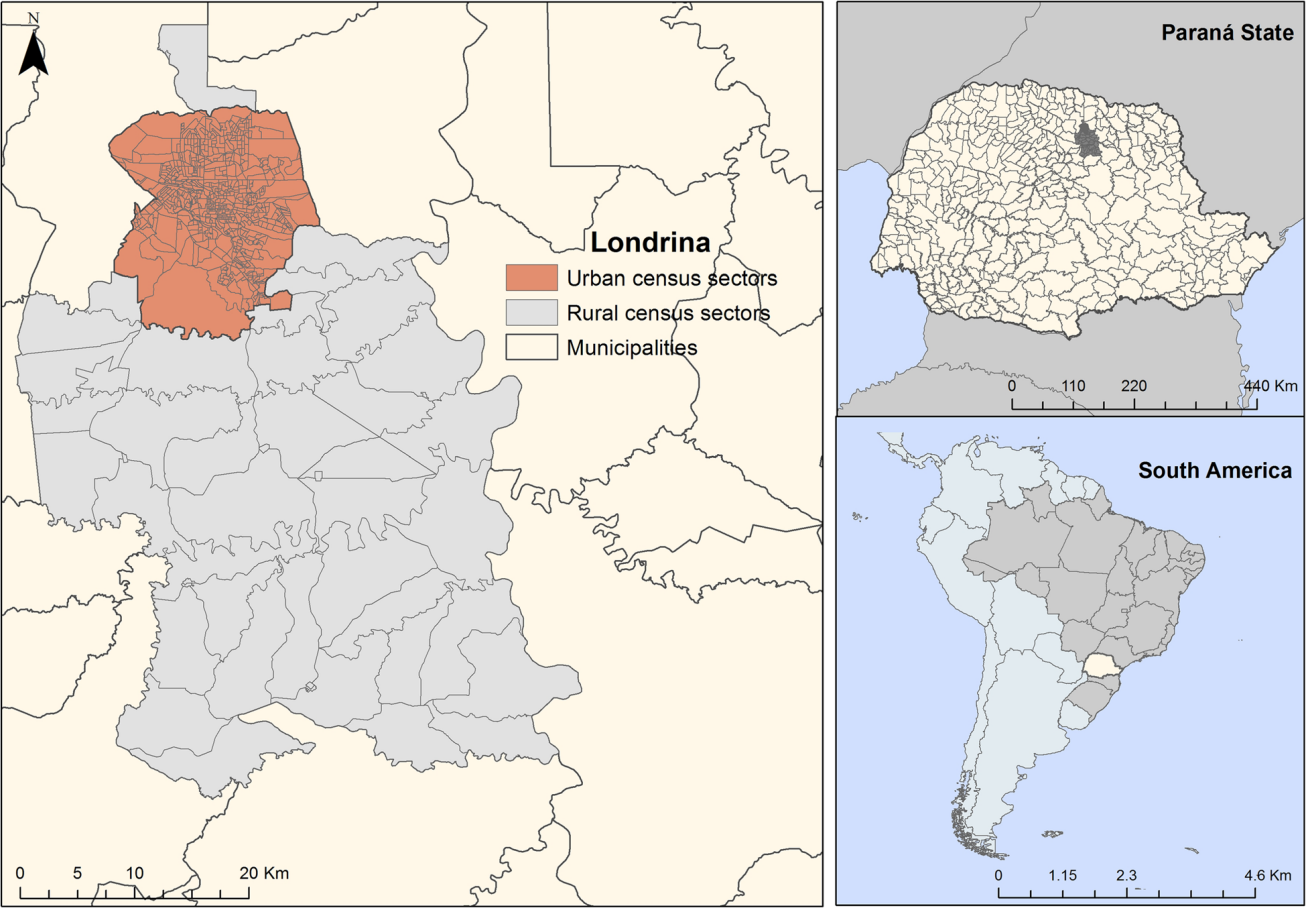
Map of the studied municipality and its geographical location. Londrina, Brazil (2008–2015)

This ecological study [10] was carried out in the municipality of Londrina, which is a centre for business, technology, research and health development [11]. It is situated at the following geographical coordinates: 23°18′ S latitude and 51°09′ W longitude [12]. Figure 1 illustrates the location of the municipality.

The municipal population is 548,249 inhabitants, of whom 493,520 are concentrated in the urban area, which is the second most populous city of Paraná. The municipal population density is 330.95 inhabitants/km^2^ [12].

In terms of social indicators, the municipality has a Municipal Human Development Index (MHDI) of 0.78, classified as a high HDI (0.700 to 0.799) according to the United Nations Development Program (UNDP) [13]. Longevity is the attribute that contributes most to the MHDI, with an index of 0.837, followed by income, with an index of 0.789, and education, with an index of 0.712. Regarding social inequality, the municipality presents a Gini coefficient of 0.42, suggesting that there is an inequality of income distribution, and a poverty rate of 36.49% [13].

The municipality has 56 primary health units (PHUs), with 86 family health strategy (FHS) teams distributed in 54 PHUs. FHS coverage is 58.62%, with 32 hospitals and two emergency care units (ECUs) [11, 12].

In 2015, Londrina had a TB incidence rate of 32.95/100,000 inhabitants, a bacillary TB incidence rate of 17.16/100,000 inhabitants and a mortality rate of 0.7/100,000 inhabitants [14].

### Study observation units

The analysis units of the study were census tracts [15]. The municipality of Londrina has 713 census tracts; the 678 tracts that are considered to be urban were used in this study.

### Study population and period

The study population consisted of cases of deaths caused by TB, with the Declaration of Death (DD) showing codes A15.0 to A19.9, according to the International Classification of Diseases (ICD version 10). This corresponded to all clinical forms of TB, and was assessed over the period from 2008 to 2015.

### Source of information and data collection

Data were obtained from two different sources. The first source was 2010 Demographic Census of the Brazilian Institute of Geography and Statistics to obtain the maps data, and the second source was the mortality information system (MIS) of the Health Surveillance Department of the Municipal Health Department of Londrina, BR. The use of the MIS is justified because it was one of the pioneering systems to be founded in Brazil [16].

### Data analysis

In the first step, descriptive statistics were performed, including the calculation of absolute frequency and proportion measures for the categorical variables and location (mean and median) and dispersion (standard deviation) measures for the continuous variables, using Statistica (v12.0) software.

Subsequently, the geographic coordinates of each address were searched using the free access Google Earth technology and the geo-referencing technique of the cases was performed using the Terraview (v4.2.2) software. The SaTScan v9.4.2 software was used to identify the risk areas for TB mortality, initially applied to the standard, and then the isotonic version [17, 18].

Scan statistics is a technique developed by Kulldorff and Nagarwalla [13]. It consists of circles that move throughout the study area around the centroids, which correspond to the centre of each territorial unit under analysis [19]. The formation of the spatial clusters is based on the calculation of the number of events found within each circle. If the observed value of the region delimited by the circle is larger than expected, it is called a risk cluster; if the value is lower than expected, it is called a low-risk or protective cluster. This procedure is repeated until all centroids are tested [20].

In the centralisation process, the log likelihood ratio (LLR) of each potential cluster is formulated based on the calculation of the mortality that is observed and expectedin and out of the circular window in which the *p*-value is assigned, according to the following formula [21]:

$$ \mathrm{LLR}=\log {\left(\frac{{\mathrm{O}}_{\mathrm{in}}}{{\mathrm{E}}_{\mathrm{in}}}\right)}^{\mathrm{O}\mathrm{in}}{\left(\frac{\mathrm{O}-{\mathrm{O}}_{\mathrm{in}}}{\mathrm{O}-{\mathrm{E}}_{\mathrm{in}}}\right)}^{\mathrm{O}-{\mathrm{O}}_{\mathrm{in}}} $$

in which O represents the observed cases and E represents the expected cases. In this way, O_in_ and E_in_ denote the number of observed and expected cases in the window, respectively. E_in_ is calculated by multiplying the deaths due to TB by the population of the census tracts. The higher the LLR, the less likely the cluster detection occurred due to chance [22].

After the formation of the cluster, the software also presents the spatial relative risk (SRR) value [23], which is obtained through an equation [24] that corresponds to the estimated risk within a cluster divided by the estimated risk outside the cluster. The SRR is calculated by taking into account the observed cases divided by the expected cases within the cluster, all divided by the observed cases divided by the expected cases outside the cluster. The equation is:

$$ SRR=\frac{N_z/{E}_z}{\left(N-{N}_z\right)/\left({E}_{A-}{E}_Z\right)} $$

where N is the total number of cases, NZ is the number of cases in the Z cluster, EA is the expected number of cases in the region under the null hypothesis, and EZ is the expected number of cases in the Z area under the null hypothesis.

The difference between the standard and isotonic methods lies in the number of circular windows generated during the centralisation procedure; the isotonic version, instead of developing only one circular window, uses a set of different sized, overlapping circles, centred on the same centroid. It also starts from the alternative hypothesis that the mortality rate is highest within the innermost circle, a little lower between the first and second circles, and so on, until the final circle [18, 24].

In the isotonic version of a given centroid, the risk is modelled as higher within some unknown distance (*d*) of the centroid compared to a greater distance from the same centroid. This means that the risk is modelled as a function *r*(*d*) of the distance from the centroid, and that it uses the steps in a risk function with a single discontinuity in *d* [25, 26].

The risk function can be classified as a non-increasing function, i.e. the greater the distance from the centroid of the territorial unit of analysis, the smaller the spatial risk for the occurrence of death due to TB. After the identification of the purely spatial risk clusters, to assess the reliability of the SRR values, the respective 95% confidence intervals (95% CI) were calculated [27].

In terms of the spatial clusters, the Poisson method was adopted, with the high and low rates option used for the two analyses, and 10, 30 and 50% of the population at risk, with circular format windows and 999 replications considered as the maximum cluster size [28, 29].

In the third step, the Gi* statistical technique was used to analyse the spatial association of TB mortality. For this, it was necessary to calculate the TB mortality rate standardised by sex and age (TxMTBi), with age classified as less than or equal to the median, or greater than the median (56 years), according to the formula:

$$ \mathrm{TxMTBi}=\frac{\Sigma\ \mathrm{of}\ \mathrm{the}\ \mathrm{standard}\mathrm{ised}\ \mathrm{by}\ \mathrm{age}\ \mathrm{X}\ \mathrm{100,000}}{\Sigma\ \mathrm{of}\ \mathrm{the}\ \mathrm{standard}\ \mathrm{population}}\ \mathrm{X}\ \frac{1}{8} $$

Next, to estimate the radius of the distance used in the Gi*, the Incremental Spatial Autocorrelation (ISA) tool provided by ArcGIS (10.5) was used. The ISA was tested for 30 distances, in which the result of the most pronounced distance was 3143.28 m with *p* < 0.01 [30, 31]. Also, for the spatial association analysis, the weight matrix normalised by distance was created using Geoda version 1.8 software.

The Getis-Ord General G, G(d), analysis was performed in ArcGIS (v.10.5) software. The G(d) consists of a global index to evaluate the spatial association of an attribute based on statistical distances and calculated from a sum of values for a given distance, according to the following formula [32]:

$$ G(d)=\frac{\sum_{i=1}^n{\sum}_{j=1}^n{w}_{ij}(d){x}_i{x}_j}{\sum_{i=1}^n{\sum}_{j=1}^n{x}_i{x}_j},j\  not\ equal\ to\ i $$

where n corresponds to the number of areas, W_ij_ is the value in the proximity matrix for region i with region j as a function of distance (d), and x_i_ and x_j_ are the values of the attributes considered in the areas i and j.

The value of G(d) is provided by a Z score, ranging from + 3 to − 3, which determines whether the attributes with high or low values are spatially grouped, with higher Z scores indicating more extreme grouping in the region, called hotspots, lower Z-scores indicating low value groupings, called coldspots, and values of 0 indicating no grouping [33, 34]. In this sense, with the intention of examining the spatial patterns in detail, the Gi* local association indicator was used. In the Gi*, the values for each location, that is, each census sector, are considered from a neighbourhood matrix.

The pseudo-significance test was used to certify the statistical validity of the results [35]. A type I error of 5% was fixed as statistically significant (*p* < 0.05) for all the tests.

### Kernel density

An estimation of the density of the nucleus was performed every year to identify whether the conglomerates were changing over time. This analysis consisted of an exploratory interpolation in which the density was generated for the visual identification of hotspot areas, which belong to regions with a higher density of TB deaths [36]. A radius of 3143.28 m was considered, according to the analysis performed through ArcGIS (v.10.5) software. Thematic maps were also constructed using this software.

### Ethical approval and consent to participate

The maps data from 2010 Demographic Census of the Brazilian Institute of Geography and Statistics are open data, and the data from the MIS were authorized of the Health Surveillance Department of the Municipal Health Department of Londrina,BR. This study was approved by the Research Ethics Committee of the School of Nursing of the University of São Paulo, Ribeirão Preto Campus, in accordance with the Guidelines and Regulatory Standards for Research with Human Subjects, Resolution No. 196/96 of the National Health Council, under Certificate of Presentation for Ethical Appreciation No. 56305516.0.0000.5393, issued on 11 December 2015. A signed consent form was not necessary as secondary data were used and the participants were not identified.

## Results

A total of 61 deaths due to TB were identified, the characteristics of which are described in Table 1. According to this table, the mean age was 56.9 years with a standard deviation (SD) of 17.8; 49 (80.3%) were male, 39 (63.9%) were white, 17 (27.9%) were married and 20 (32.8%) had a high school education. According to Table 1, 32 (52.4%) presented the pulmonary clinical form of TB. All identified cases were georeferenced, with a general municipal mortality rate of 1.5 cases per 100,000 inhabitants.

**Table 1:** Characteristics of the individuals who died of tuberculosis in Londrina, Brazil (2008–2015)

Variable	n	%
Age (years)		
< 19	1	1.6
20–39	8	13.1
40–59	27	44.3
≥ 60	25	41.0
Median	56	
Mean	56.9	
Gender		
Male	49	80.3
Female	12	19.7
Skin colour/ethnicity		
White (Caucasian)	39	63.9
Black (African)	9	14.8
Yellow (Asian)	4	6.6
Brown (Mixed race)	9	14.8
Marital status		
Single	17	27.9
Married	23	37.7
Widowed	6	9.8
Divorced	6	9.8
Steady partner	2	3.3
No response	7	11.5
Level of education		
No schooling	1	1.6
Elementary education	12	19.7
High school education	20	32.8
Higher education	15	24.6
No response	13	21.3
Occupation		
Retired/pensioner	13	21.3
Homemaker	7	11.5
Miscellaneous	28	45.9
No response	13	21.3
Place of death		
Hospital	54	88.5
Home	7	11.5
Received medical assistance		
Yes	32	52.5
No	2	3.3
No response	27	44.3
Diagnosis confirmed by further examination		
Yes	14	23.0
No	2	3.3
No response	45	73.8
Diagnosis confirmed by surgery		
Yes	1	1.6
No	15	24.6
No response	45	73.8
Diagnosis confirmed by necropsy		
Yes	5	8.2
No	36	59.0
No response	20	32.8
Death certified by		
Assistant	17	27.9
Substitute	19	31.1
Death Surveillance Service	2	3.3
TB site		
Pulmonary	32	52.4
Extra-pulmonary	29	47.5

Source: *MIS* Londrina, *BR* 2016

**Fig. 2:**
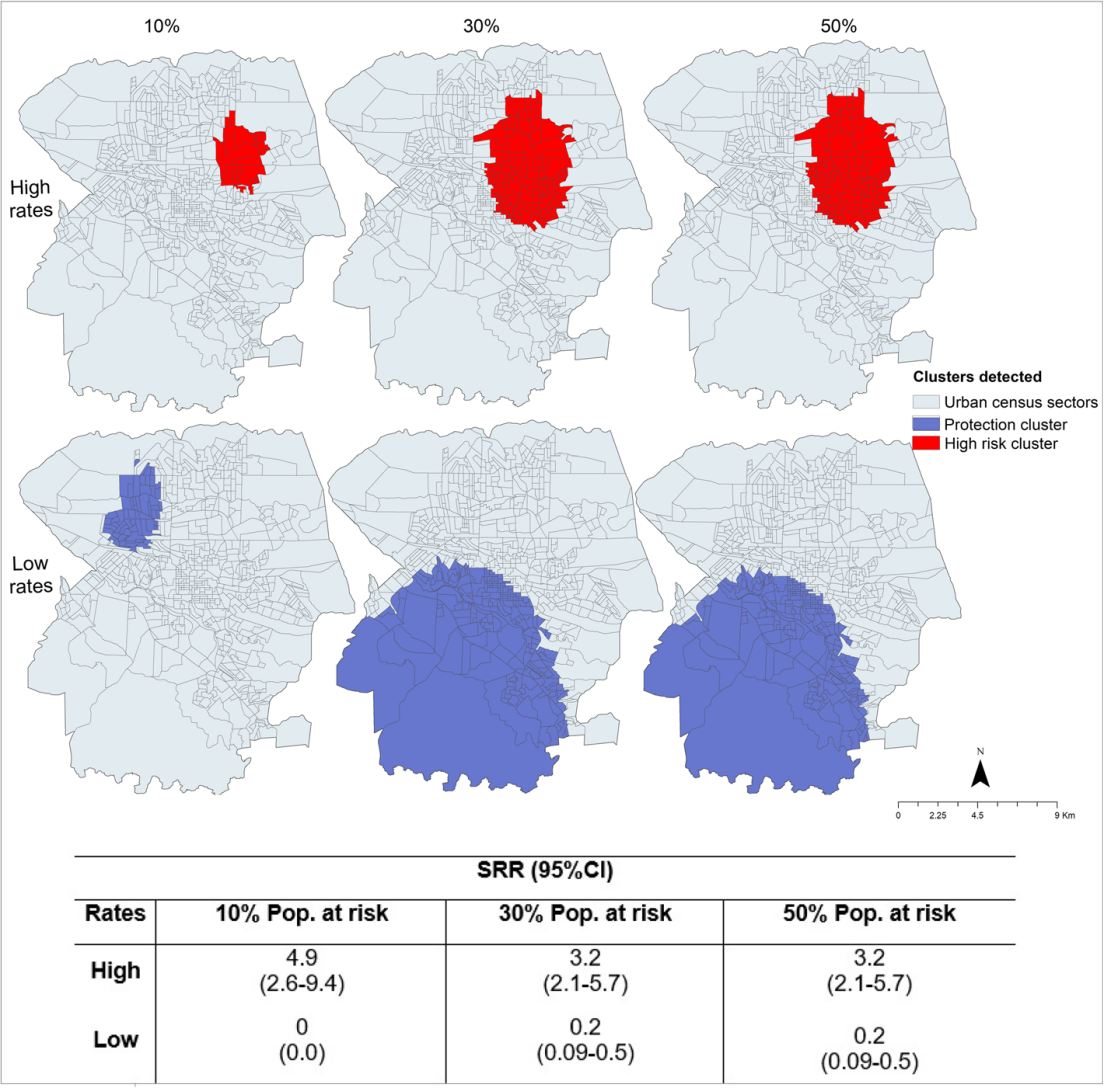
Clusters of high-low risk for TB mortality according to the exposed population, Londrina, Brazil (2008–2015)

Regarding the application of the standard for high rates, a risk cluster was identified for each percentage of the population exposed, i.e. 10, 30 and 50%, with the SRR and 95% CI values expressed in Fig. 2.

In Fig. 2, the clusters of risk covered the north and east regions of the municipality, with 12 deaths in the cluster with 10% of the population at risk and 29 deaths for 30 and 50%. Also, usingk the traditional scan, for low rates and 10% of the population exposed, a protection cluster was identified in the northwestern region of the municipality, where there were no deaths due to TB, whereas for 30 and 50% of the population at risk, the protection cluster involved the entire southern region of the municipality, with 5 deaths for each cluster.

**Fig. 3:**
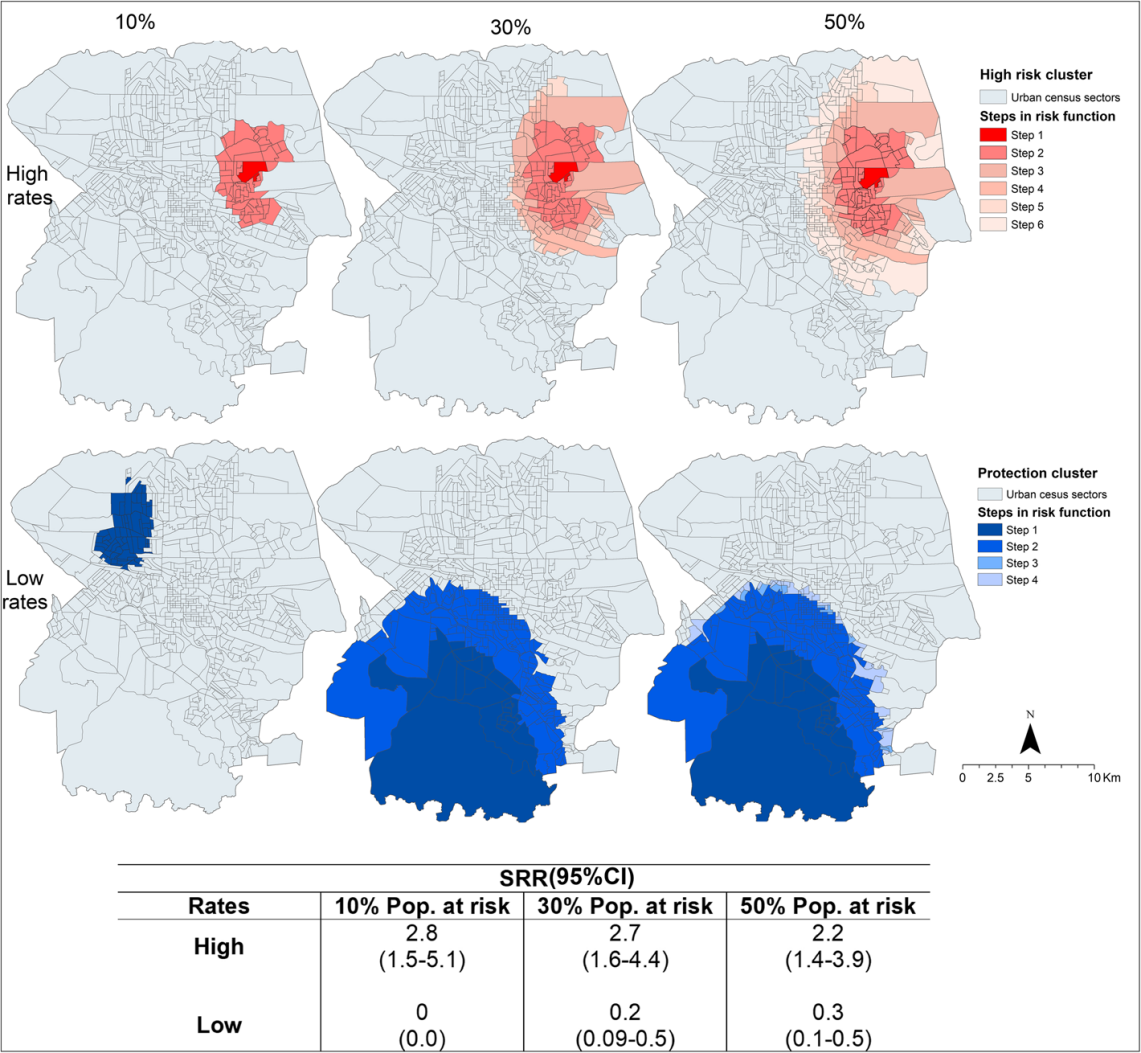
Clusters of TB deaths in the exposed population, identified through isotonic spatial scan statistics, Londrina, Brazil (2008–2015)

Referring to the isotonic version, as seen in Fig. 3, spatial risk clusters were also found in the northern and eastern regions of the municipality, demonstrating that there was no change in the location of the risk clusters. The protection clusters also maintained the same location as in the traditional scan. The SRR and 95% CI values are described in Fig. 3.

In the isotonic version, the steps in the risk function are present, which corresponds to the multiple circular windows formed during the centralisation process of the scan. This also maximises the LLR. These steps allowed for the verification of the intensity of mortality within the clusters. Thus, for the risk cluster with 10% of the population, step 1 had the lowest radius (0.3 km; Table 2) and the highest SRR value (20.8; 95% CI 7.5–57.1), totalling 4 deaths, whereas step 2 had a radius of 1.8 km and a SRR of 2.05 (95% CI 1.1–3.0), totalling 14 deaths.

**Table 2:** Risk clusters for TB deaths, according to the steps in risk function, in Londrina, Brazil (2008–2015)

Rate	Pop. at risk (%)	Steps in risk function	No. census sectors	Pop. of cluster	Annual cases/100,000	No. Cases	Expected cases	SRR^a^ (95% CI)	Radius (km)
High	10	1	2	1846	27.0	4	0.2	20.8 (7.5–57.1)	0.3
		2	62	47193	3.7	14	5.7	2.05 (1.1–3.7)	1.8
	30	1	2	1846	27.0	4	0.2	24.4 (8.9–67.3)	0.3
		2	71	55843	3.5	16	6.8	2.43 (1.4–4.3)	1.9
		3	107	79956	3.2	21	2.9	2.31 (1.3–3.9)	2.5
		4	130	95969	3.1	24	3.6	2.09 (1.2–3.4)	2.8
		5	149	112273	3.0	27	2.0	2.03 (1.2–3.3)	2.9
	50	1	2	1846	27.0	4	0.2	24.46 (8.9–67.3)	0.3
		2	71	55843	3.5	16	6.8	2.43 (1.4–4.3)	1.9
		3	107	79956	3.2	21	2.9	2.31 (1.3–3.9)	2.5
		4	130	95969	3.1	24	1.9	2.09 (1.2–3.4)	2.8
		5	149	112273	3.0	27	2.0	2.03 (1.2–3.3)	2.9
		6	281	203846	2.3	38	11.7	1.4 (1.3–3.9)	3.9
Low	10	1	63	53032	0	0	6.1	0 (0.0)	1.6
	30	1	31	23448	0	0	2.7	0 (0.0)	4.0
		2	216	35782	1.7	5	15.5	0.25 (0.1–0.6)	6.0
	50	1	31	23448	0	0	2.7	0 (0.0)	4.0
		2	217	144348	0.4	5	15.6	0.23 (0.09–0.6)	6.0
		3	230	154346	0.5	6	1.3	0.6 (0.1–0.5)	6.0
		4	261	156194	0.6	8	2.3	0.63 (0.1–0.6)	6.3

^a^Spatial relative risk

The risk cluster for 50% of the population at risk had six steps, with step 1 having a radius of 0.3 km and SRR of 24.4 (95% CI 8.9–67.3), with 4 deaths, and step 6 having a radius of 3.9 km and SRR of 1.4 (95% CI 1.3–3.9), with 38 deaths. In this way, the smaller the cluster radius of the risk clusters, the higher the SRR value.

Regarding the protection clusters, step 1 had the lowest SRR, as it occurred in the cluster for 30% of the population at risk, in which step 1 presented a radius of 4.0 km and SRR of 0 and step 2 had a radius of 6.0 km and SRR of 0.25 (95% CI 0.1–0.6), totalling 5 deaths, i.e. the smaller the radius, the smaller the SRR value.

Table 2 shows the spatial characteristics of TB deaths within the clusters identified through the isotonic version according to the steps in risk function.

Table 3 compares 346 the data obtained through the standard scan and the isotonic scan.

**Table 3:** Comparative analysis of standard and isotonic scans of TB mortality, Londrina, Brazil (2008–2015)

Rate		High			Low		
Pop. at risk (%)		10	30	50	10	30	50
No. census sectors	Standard	32	153	153	63	216	217
	Isotonic	60	149	281	63	216	261
Pop. of cluster	Standard	22993	102433	102433	53032	143762	144328
	Isotonic	47193	112265	203830	53032	143762	172652
Annual cases/100,000	Standard	6.4	3.4	3.4	0	0.4	0.4
	Isotonic	3.7	3.0	2.3	0	0.4	0.6
No. cases	Standard	12	29	29	0	5	5
	Isotonic	14	27	38	0	5	8
Expected cases	Standard	2.9	13.3	13.3	6	18.3	18.4
	Isotonic	5.9	14	25.8	6.1	18.3	22
Radius (km)	Standard	1.4	2.4	2.4	1.6	5.9	6.0
	Isotonic	1.8	2.9	3.9	1.6	5.9	6.3
LLR^a^	Standard	8.7	9.7	9.7	6.4	8.7	8.7
	Isotonic	9.6	12.3	12.7	6.4	9.5	9.9
*p*-value	Standard	0.05	0.02^**^	0.03^**^	0.05	0.04^**^	0.04^**^
	Isotonic	0.04^**^	0.01^**^	< 0.01^**^	0.05	0.02^**^	0.02^**^

^a^Log likelihood ratio; ^**^p < 0.05

Table 3 shows that the isotonic version covered more census tracts due to the larger radius dimensions. For 10 and 50% of the population, there were 60 and 281 sectors in the isotonic versus 32 and 153 sectors in the traditional version, respectively.

In terms of TB cases, the traditional version captured fewer cases than the isotonic version, with the traditional version presenting 29 deaths for 50% of the population, compared to 38 deaths in the isotonic version. The SRR in the traditional version remained larger than that in the isotonic version, ranging from 3.2 to 4.9 and 2.2 to 2.8, respectively; however, in the isotonic version, the SRR assumed higher standards within the steps, ranging from 1.4 to 24.4.

A similar case occurred for the mortality rates, with the traditional version calculating 3.4 to 6.4 deaths per 100,000 inhabitants, whereas the isotonic version calculated 2.3 to 3.7 deaths per 100,000 inhabitants. However, the rates within the steps ranged from 3.0 to 27.0 deaths per 100,000 inhabitants.

For the protection clusters, the data were similar when considering 10 and 30% of the population at risk, however, when the size of the cluster was changed to 50% of the population, the isotonic version grouped more census sectors, with 261 sectors and more TB deaths, totalling 8. An important aspect to be emphasised is the LLR values; as Table 3 shows, in the isotonic version, the LLRs were increased for both the risk and protection clusters, highlighting that the randomness of the clusters in the isotonic version was lower than in the traditional version.

**Fig. 4:**
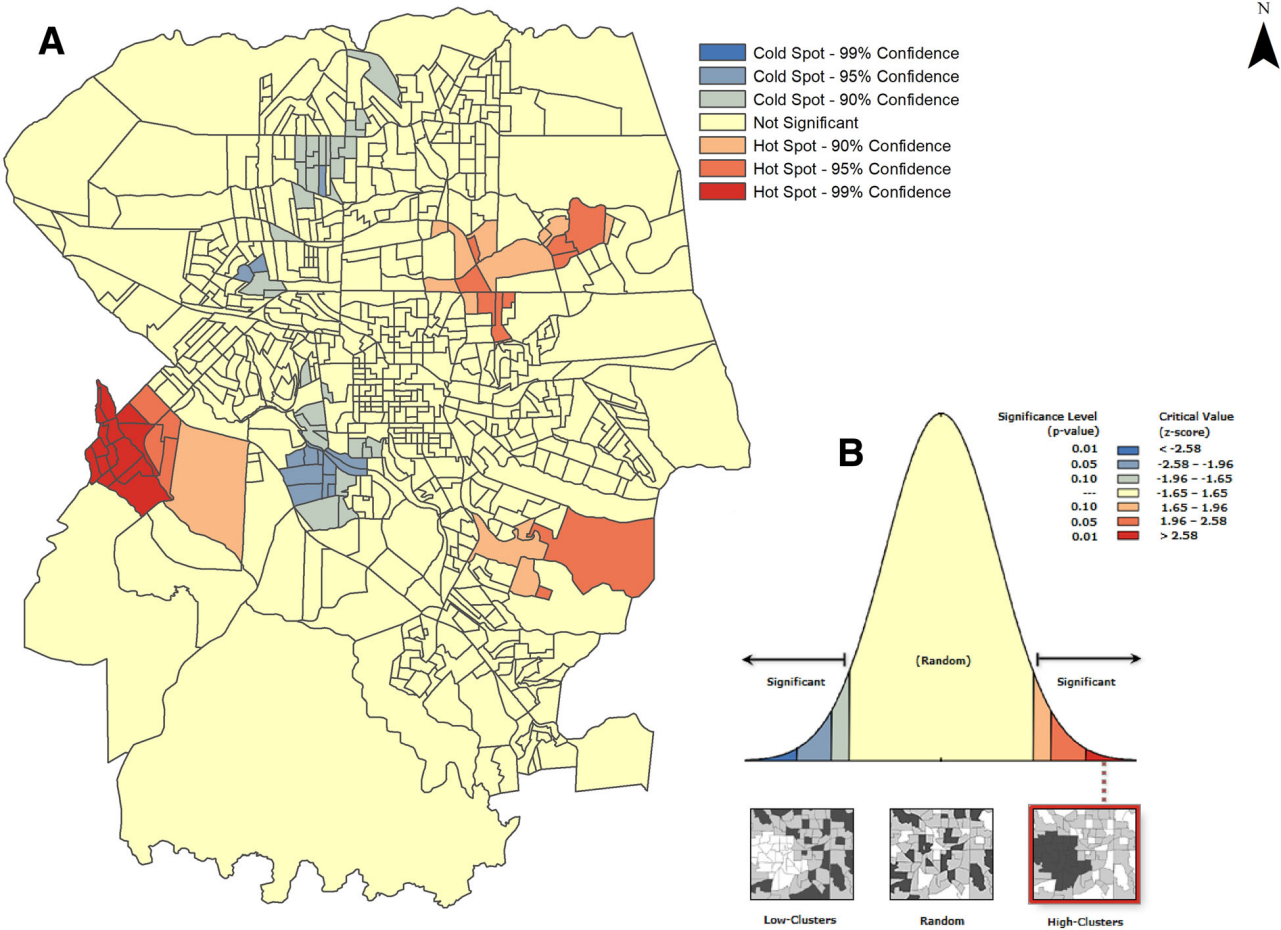
Hotspots and coldspots related to the risk of TB mortality, according to Gi*, Londrina, Brazil (2008–2015). **a** Gi* local spatial association **b** Level of statistical significance of the General G statistics

Regarding the spatial association, Fig. 4 shows the hotspots and coldspots detected by the global and local analysis of the Getis-Ord statistic. The general index of the Getis-Ord statistic (Fig. 4b) had a value of 0.002, demonstrating a positive spatial relationship. The local analysis Gi* (Fig. 4a) demonstrated the formation of hotspots in the eastern regions, with 95% confidence, and in the western region, with 99% confidence, where the Gi Z-score was higher than 1.96. Coldspots were also found in the northwestern and southern regions of the municipality, with 95% confidence level. The pseudo-significance test (Fig. 4b) confirmed both the statistical significance of the analysis, with *p* < 0.01, and the non-randomness of the clusters.

**Fig. 5:**
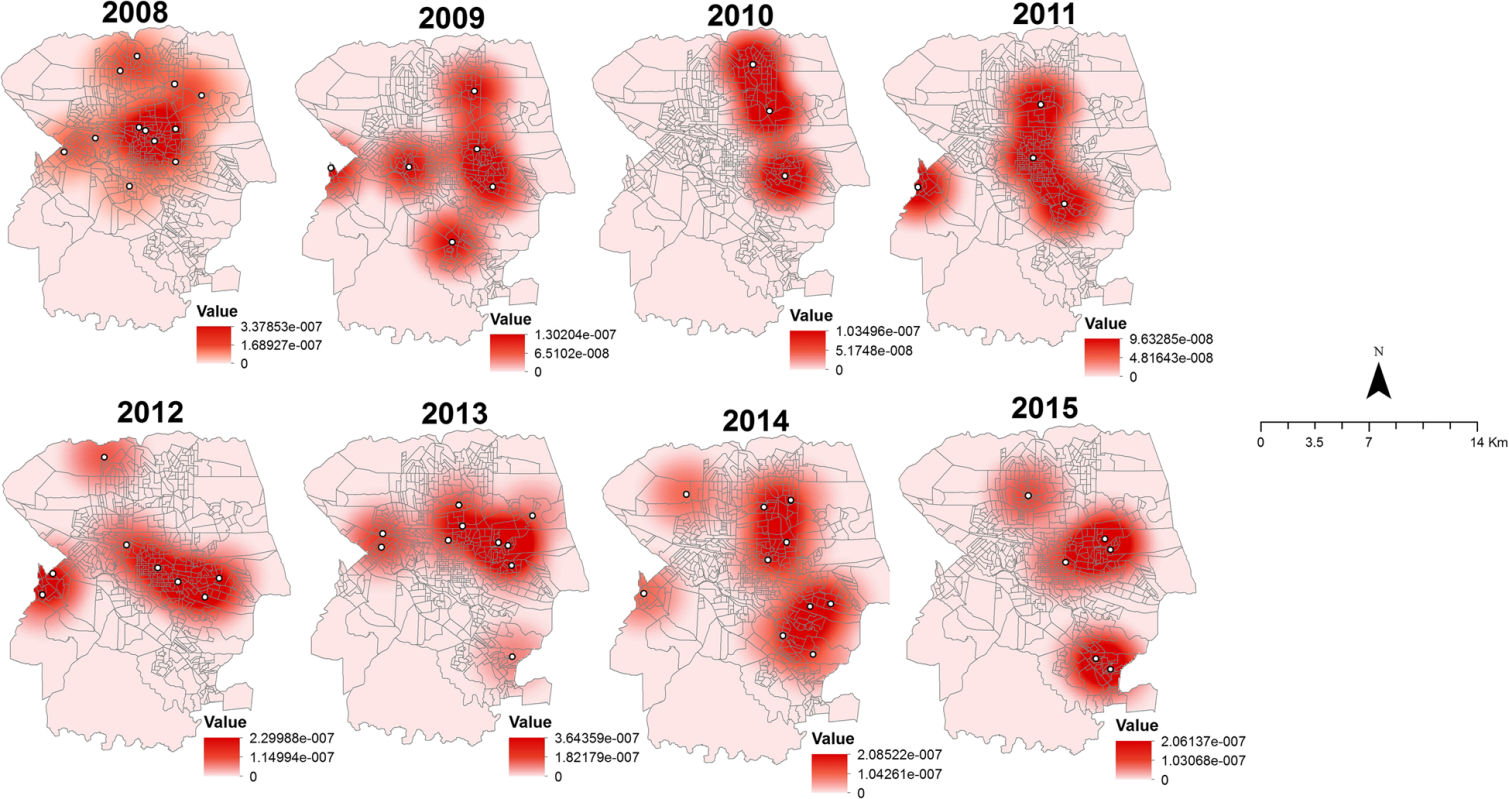
Spatial and temporal distribution of TB mortality according to the Kernel density, Londrina, Brazil (2008–2015)

Figure 5 shows the distribution of the highest density of deaths due to TB for each year of this study.

## Discussion

This study aimed to identify risk clusters for TB deaths, specifically in areas where TB supposedly does not seem to be a problem. We observed that the isotonic version covered a greater area of risk than the traditional method, areas that would be considered to be non-problematic or unknown by health care services.

The difference between the traditional method and the isotonic version consists of the number of circular windows generated during the centralisation procedure. The isotonic version, instead of developing only one circular window, uses a set of different sized, overlapping circles, centred on the same centroid. It also starts from the alternative hypothesis that the mortality rate is highest within the innermost circle, a little lower between the first and second circles, and so on, until the final circle [18]. Despite the isotonic version evaluating a window with multiple circles, only a single circle provides the highest likelihood and therefore defines the most probable cluster; in this way, the isotonic version and Gi* proved to be more suitable spatial techniques to analyse territorial subunits and with rarer events.

When Gi* was performed, a new hotspot region was identified, which would have been unknown or underdiagnosed by scan statistics. Although this technique does not follow the same analysis pattern as scan statistics (Fig. 4), it diagnosed clusters in areas where TB was supposedly not a problem.

These findings are important to keep in mind when considering the procedures and tools that have been used by health surveillance and health care workers. When the results are negative, it is likely that these areas will not be prioritised in terms of active TB case finding, the education of health professionals and community, and investigations into health care services, among other actions [37, 38]. When risk clusters for TB deaths are identified, these areas might be prioritised in terms of new resources and investment to improve health conditions and access [6, 7]. thus advancing health equity. The results might motivate integration between epidemiological surveillance activities and Primary Health Care and this way, early diagnosis, the active search for respiratory symptomatology, and the follow-up of patients.

Due to the development of information technology, epidemiology has added a series of modern instruments to its arsenal, which allow for the consideration of the issue of space and to deepen reflections on social relations and the conditions that differ between the risk of dying and living in the different areas under investigation; this study has produced important contributions to these areas.

In relation to the profile of the deaths investigated in the study, the most affected age group corresponded to the economically active population, corroborating the findings in the literature [39, 40]. Another important finding was that, in spite of the predominance of middle-aged deaths, deaths in older adults were also highlighted as, due to the increased longevity of the population, the municipality is facing an increasing number of older adults with TB. Senility has been found to be a risk factor for TB disease [41–43]. Males were more frequently affected by death, as was the case in the studies by Dale [44], Beyene [45] and Ferrer [46], which associated this fact with the cultural, economic and social factors related to TB exposure.

Studies show that TB mortality in Brazil occurs predominantly in the non-white population [9, 47, 48]. However, in the present study, the white population was most affected. This was probably due to the fact that the municipality was colonised by Europeans and 70% of the population identifies as white [26, 49]. The majority of cases had a complete high school education, unlike the findings in the literature, which report that the level of schooling is an essential tool for understanding TB patients in terms of their clinical situation and adherence to treatment, with increased education reducing the index and risk of death [50–52].

Regarding occupation, retirees/pensioners were prevalent. According to Cavalcante [53], this is due to the fact that retirees present greater vulnerability in terms of economic conditions, difficulties in transportation to health services, abandonment by family members and the difficulties in implementing public policy that meet the health demands of older adults.

The majority of the deaths occurred in the hospital after receiving medical care, with this finding highlighting difficulties in the early diagnosis of TB and the consequent delay in the treatment of these patients; this may be due to shortcomings in the primary healthcare system. It is important to highlight that the coverage of the FHS is approximately 58% in the municipality [53–56].

This study showed the pulmonary form of TB to be the most frequent in cases of death, which has also been observed in other studies [44, 50]. However, there was no bacteriological or histological confirmation information (ICD A16.2) for the classification of death.

Regarding the methods of analysis used to identify the risk of mortality in the areas under study, both techniques showed spatial risk and protection clusters for the occurrence of death due to TB (Figs. 2, 3 and 4) in the same regions of the municipality, but with different conformations. The isotonic analysis produced spatial risk clusters with larger dimensions, grouping more census tracts and calculating a greater number of deaths, compared to the standard version. This finding corroborates a study carried out by Kulldorff [18] on breast cancer in the United States.

As highlighted in the results of the study, a spatial scan detects the general location of the cluster by treating the SRR as a constant value, whereas the isotonic version presents the steps in risk function, which stratifies the radius sizes and SRR values within the cluster. This allows for a better diagnosis of the mortality in the municipality and shows areas with real risks of the event or those that constitute regions of protection.

In addition to the discussion of protection clusters, which were identified by both analyses (traditional and isotonic) and in the Gi* analysis, large differences were not observed in terms of the protection areas when the approaches were compared. Specifically regarding the protection areas shown by the study, it should be mentioned that the results were derived from data generated by health services and, therefore, their existence may be due to the underreporting of TB, although MIS is considered the gold standard throughout the country.

Through the kernel density analysis, there was little variation in the spatial distribution of deaths over time. Moreover, the areas of risk identified in the other spatial techniques remained the same, highlighting the eastern region, which continued to have a high density of deaths in all study years.

Mortality from tuberculosis can be influenced by the degree of integration between epidemiological surveillance activities and care provided over time, especially with regard to primary health care. Changes in health policies that do not focus on strengthening primary health care are commonly observed in Brazil. Therefore, mortality rates are also influenced by health actions, such as the training of health professionals to perform early diagnosis, the active search for respiratory symptomatology, and the follow-up of patients. In addition, local socioeconomic inequalities also suffer from variations in space-time, directly related with TB mortality.

This study worked with a small number of observations, since death due to TB is an infrequent phenomenon in the region. Thus, using traditional scan statistics might result in false negative areas of risk, which could compromise the surveillance actions of the city.

Since this study introduced alternative methods for diagnosing risk clusters and confirmed areas that were considered to be non-problematic, this study can become a reference for other small Brazilian municipalities (about 5,000 cities) where TB mortality may still be occult or silent. The application of the Gi* and kernel methods is more frequent than the isotonic spatial scan statistic, yet, when they are used together, the results become much more consistent, reliable and valid.

A reduction in TB mortality requires an understating of the dynamics of TB at the local level, not only at the national or state levels. Since most Brazilian municipalities are small in size, new approaches, including geo-statistics, are absolutely necessary to unravel some mysteries. Stevens and Pfeiffer [57] corroborate this idea by emphasising that innovative methodologies for a more congruous spatial approach to health enable the discovery of results that are more in line with the real situation and can provide guidance for health decision making.

The authors understand that the study might be a reference for diagnosing risk TB mortality and others rarer diseases, as well as a tool to guide policy makers, managers, stakeholders in the allocation of resources, such as cash transfers (Bolsa Família) and others social pockets according to the areas of highest risk [1–3].

The limitations of this study include the use of secondary data, which may have introduced bias due to the presence of gaps or incomplete data. It should also be noted that for the spatial analyses, only the cases of the deaths of individuals living in the urban area of the municipality were considered.

## Conclusions

In conclusion, risk areas for tuberculosis mortality have been identified, including areas where TB was supposedly not a problem. These findings were achieved due to the combination of complementary methods. This study does not allow for a conclusion in terms of the best method for estimating the spatial risk for small regions, since the concepts and approach of each method are different. For this, a different study design would be necessary, with a focus on geostatistical analysis through simulations and more dense and robust tests. These findings show the need to match methods to confer more accurate results, specifically in areas where the phenomenon is rare.

## Data Availability

Mortality data that were the basis for this study belong to the Department of Epidemiology of the Health Secretariat of Londrina.

## Abbreviations

DD: Declaration of Death
FHS: Family Health Strategy
Gi*: Getis-Ord Gi* statistics
ICD: International Classification of Diseases
MHDI: Municipal Human Development Index
MIS: Mortality Information System
PHUs: Primary Health Units
TB: Tuberculosis
WHO: World Health Organization
